# Biology of the avian respiratory system: development, evolutionary morphology, function and clinical considerations

**DOI:** 10.1098/rstb.2023.0419

**Published:** 2025-02-27

**Authors:** John N. Maina, Emma R. Schachner

**Affiliations:** ^1^ Department of Zoology, University of Johannesburg, Johannesburg 2006, South Africa; ^2^ Department of Physiological Sciences, College of Veterinary Medicine, University of Florida, Gainesville, FL 32608, USA

**Keywords:** birds, diversity, lung, pulmonary, evolution, skeleton

## Abstract

The respiratory biology of birds has been of interest to researchers for centuries, particularly owing to its dramatically heterogeneous structure, unusual ability for non-ventilatory structures to invade nearly all parts of the body (including the skeleton) in many taxa, and its exceptional efficiency under high-altitude hypoxia. Advances in imaging, experimental and developmental techniques, as well as recent palaeontological specimens have facilitated new discoveries, analyses and progress in the field. Comprehensively, this theme issue shows the origin of the modern avian respiratory system, current controversies and how the evolution of respiratory structures in birds has impacted their biology from the molecular, to the cellular, to the phylogenetic level. This collection of articles addresses progress the field has made, gaps in our knowledge and where the field needs to go, with a primary focus on adult and embryonic form and function but also touching on vocalization and clinical aspects of avian respiratory biology.

This article is part of the theme issue ‘The biology of the avian respiratory system’.

## 1. Introduction

Avian populations are declining worldwide, with some sources indicating that their numbers have diminished by 30–40% in some groups relative to the 1970s [1]. With climate-change-driven habitat loss and transformation of landscapes by human activity as leading causes, interactions between birds and their environment from an ecological perspective are a popular area of study [1,2]. The respiratory system of birds is the primary site of gas exchange, and thus a direct intersection between these animals and the environment [3]; however, much of the respiratory biology of birds remains poorly resolved [4], and this topic is, in our opinion, understudied relative to other aspects of avian biology and deserving of greater attention. Presently, articles on avian respiratory biology are scattered across journals, books and myriad other sources. The last noteworthy comprehensive compendium of treatises on the biology of the avian respiratory system was from a conference organized by Hans-Reiner Duncker in 1977, held at the Max-Planck-Institute for Experimental Medicine, Göttingen (FRG), 28−30 July. The Proceedings of the meeting were later published in a book entitled *Respiratory function in birds, adult and embryonic* [5]. Since then, over the nearly five decades that have elapsed, considerable advances have been made in the field, but they have been scattered and sporadic. To the best of our knowledge, no major symposium or conference addressing the subject matter has occurred, with only one book compiling contributed papers, which was much smaller and narrower in scope [6].

There have been substantial advances in imaging and anatomical modelling techniques, developmental technologies and relevant palaeontological discoveries impacting avian respiratory biology. As a result, it is time for a new update of the ‘state of the art’ research that has been conducted over the last 10 years from current leaders in the field as well as new early career researchers. Additionally, there are some hotly debated topics on the evolution of the avian respiratory system and postcranial pneumaticity that we wish to bring to light. It is our aim that this special issue serves to reignite the kind of interest in avian respiratory biology that thrived in the 1970s, similar to the ongoing anatomical and developmental interest in the vertebrate skull and face [7]—a renaissance in the field, so to speak. This special issue has the overarching broad theme of addressing topics from the complete range of avian respiratory biology: from embryonic development to the adult form, including fossil data, neuroscience, physiology, and veterinary medicine topics.

## 2. Functional anatomy and physiology

Despite the respiratory system being one of the critical ways an organism interacts with the external environment, the lungs are often relegated to the background as a research topic unless it is under the umbrella of human clinical research. By providing new data and emphasizing the areas where information is lacking, our goal with the contributions in this theme issue is to motivate, advance and broaden investigations on the biology of the avian respiratory system. This special issue contains eight manuscripts addressing the form, function and physiology of the avian respiratory system, including from an evolutionary and comparative perspective. Specifically, Maina [8] provides a broad review of pulmonary structure and function, while Schachner & Moore [9] address the various aspects of the avian respiratory system that differentiate it from their sauropsid relatives and current outstanding controversies. Klein *et al*. [10] provide a broad analysis of air sac morphology and evolution, and Martinez *et al*. [11] present a micro-computed tomography (micro-CT)-derived three-dimensional digital model of a complete passerine lung and make quantitative comparisons with parrots. Adam *et al*. [12] use synchrotron X-ray CT imaging to describe new muscles in the syrinx of the zebra finch. Goller [13] provides an analysis of the latest work on the relationship between respiration and birdsong, and which questions remain controversial and needing attention. Lague *et al*. [14] address the physiological challenges that living at a high altitude imposes on the avian respiratory system, and Ponganis *et al*. [15] describe the anatomy and physiology of diving penguins.

## 3. Postcranial pneumaticity and palaeontology

One of the most notable traits of the avian respiratory system is the presence of postcranial pneumaticity, the capacity of pulmonary tissues to invade skeletal tissues beginning at approximately two months post-hatching [16]. This is a very poorly studied phenomenon; however, four articles are dedicated to postcranial pneumaticity and palaeontological specimens in this special issue. Moore & Schachner [17] provide a broad overview of pulmonary tissues invading the postcranial skeleton in birds and their non-avian dinosaurian relatives, and address mechanistic functional hypotheses for this trait. Burton *et al*. [18] quantify estimated versus true air space proportion in deceased birds and find that true air space is lower and warn of overestimating volume fraction of pneumatic bones in skeletal density studies. Gutherz *et al*. [19] provide a detailed imaging-based analysis of the post-hatch pneumatizing process of the humerus in the domesticated turkey and reveal a complex dynamic relationship between the pulmonary tissues and both bone and marrow. O’Connor [20] provides more of a deep time perspective, with data on fossil birds, including *Archaeopteryx*.

## 4. Development and clinical relevance

This special issue also includes articles on avian development and with clinical relevance. Burggren *et al*. [21] give a broad overview of the use of avian embryos in physiological and other biomedical research—an update on the current state of the field. Choi *et al*. [22] provide an experimental study on zebra finches demonstrating how this model system responds to environmental changes during embryonic development, specifically the impact of hypoxia and elevated temperature on hatching rates and respiration. Mthombeni *et al*. [23] describe the development of the chorioallantoic membrane of the common ostrich (*Struthio camelus australis*), determining that there were two distinct growth phases. Kunchala *et al*. [24] provide an analysis of the surfactant proteins in the lungs of birds, which is particularly relevant in light of the current avian influenza outbreak. Last, Ludders [25] offers a review of anaesthetic delivery and management in birds; the anatomical and physiological aspects of avian biology that make this particular aspect of avian medicine particularly challenging are underscored.

## 5. Concluding remarks

The compilation of papers in this special issue provides a solid reference base for future research in the area of avian respiratory evolutionary biology, including everything from metabolic respiration in the egg and lung development, to the lung–bone interface in fossil taxa, and three-dimensional digital models of lungs in finches. It is our hope that the most current evolutionary, developmental, morphological and physiological accounts that form this theme issue will stimulate future ground-breaking studies. We invited manuscripts that include cutting-edge research methodology, e.g. three-dimensional anatomical modelling, and novel developmental and physiological techniques. While the unifying theme of this proposal is avian respiratory biology, the data provided are far reaching; we believe that the data and details will be useful to a much larger audience interested in avian evolution, organ system biology, skeletal morphology, palaeontology, ecology, avian medicine, comparative morphology, neuroscience, vertebrate locomotion, development, modelling and three-dimensional reconstruction, and three-dimensional printing for teaching and outreach. Indeed, the three-dimensional datasets from some of the contributions are available freely online via public databases [9,11]. Many of the review contributions are critical syntheses of their subject area and will allow the reader to holistically and heuristically better understand the issues, including important future areas of study, and prevailing controversies.

## Data Availability

This article has no additional data.
